# Simulation-Based Analysis and Optimization of High-Performance Dielectric Strength Polymers in the Injection Molding of Electrical Connectors

**DOI:** 10.3390/polym17182465

**Published:** 2025-09-12

**Authors:** Fuat Tan

**Affiliations:** Department of Mechanical Engineering, Balikesir University, 10145 Balikesir, Turkey; fuattan@balikesir.edu.tr

**Keywords:** electrical connector, simulation, injection molding, process optimization

## Abstract

In this research, the thermal and structural responses of high-performance dielectric strength polymers in the injection molding process for multi-pin electrical connectors were thoroughly studied using Moldflow simulations and optimized via a Box–Behnken experimental design under the Response Surface Methodology (RSM). Injection molding analyses were performed on Polyether-ether-ketone (PEEK), Polyetherimide (PEI), and Polyamide-imide (PAI) polymers using the MS3102A 16S-1P electrical connector model. In the conducted simulations, the melt temperature, injection time, and mold open time were evaluated as three fundamental process parameters through multivariate analysis. The volumetric shrinkage, sink mark depth, residual stress, warpage, and surface temperature homogeneity were considered as the major output qualities. According to the results, the PAI material provided superior thermal stability with an average heat removal capacity of 0.127 kW, whereas the PEI material exhibited the most homogeneous cooling behavior with a surface temperature of 45.5 °C. The minimum warpage was found to be 0.254 mm, whereas the sink mark depth was recorded within the range of 0.018–0.031 mm and the rate of volume shrinkage was between 1.03% and 1.41% in the investigations. The PAI material gave the maximum residual stress of 81.9 MPa in oriented regions of the mold. This study fills a considerable gap in the field by investigating material choice and process parameter adjustments via multivariate analysis, particularly for decision making in the production of high-reliability electrical components.

## 1. Introduction

Multi-pin electrical connectors are trusted to deliver highly reliable performance; hence, they are used in defense, aerospace, industrial automation, heavy-duty vehicles, and electronic systems, which require such performance. The manufacturing of parts subject to these conditions necessitates that they not only resist high temperatures and harsh environmental conditions, such as impact, humidity, and vibration, but also have the ability to provide electrical insulation and maintain structural integrity for a long time. Therefore, engineering polymers with high dielectric strength, rather than conventional thermoplastics, should be taken into consideration. Due to their complicated geometries and tight tolerances, manufacturing and quality control should be performed with significant care and precision for connectors of this type. The process of creating such connectors via the injection molding method involves a number of obstacles due to the fact that the materials used are highly viscous, have a narrow thermal processing window, and are very sensitive to temperature changes. Furthermore, the very structure of the multi-pin leads to cooling imbalances, sink marks, residual stress, and warpage, which are typical quality problems. Therefore, a thorough simulation study is necessary at the beginning of the production process to adjust the mold design in a proper way and to increase the reliability of manufacturing. In the literature, injection molding simulation has been widely applied to polymer parts for the automotive, home appliance, and packaging sectors. However, although some studies have addressed the plastic injection process for electrical connectors, no study has directly examined injection molding and the use of dielectric polymers in multi-pin connectors. Thus, special multidimensional analyses of the moldability of high-strength engineering polymers such as PAI, PEI, and PEEK, which require high thermal resistance and superior electrical insulation capacities, are urgently needed.

The quality of injection-molded parts is primarily influenced by the material properties, the mold structure, and the parameters of the molding process. To achieve high appearance quality and accurate dimensional control, it is vital to adjust the process parameters that influence the injection molding quality. In the last decade, thermoplastic polymers have been increasingly gaining ground in the field of high-performance structural materials for various applications due to their low weight, good mechanical properties, and short cycle time in injection molding processes [1,2]. As the molds, machines, materials, and products are all chosen before processing, the molding process parameters must be carefully adjusted to not only improve efficiency but also avoid or reduce the quality defects, energy consumption, and costs associated with the process [3]. Earlier investigators employed the Taguchi method and analysis of variance (ANOVA) to explore the impacts of warpage and shrinkage on product characteristics [4]. Oktem et al. [5], Tang et al. [6], Kurt et al. [7], and Shi et al. [8] combined neural networks (NNs) and genetic algorithms (GAs), using multi-layer neural networks to estimate the maximum shear stress and GAs to find the best molding process parameters. Ozcelik et al. [9] first chose key process parameters via ANOVA and then employed NNs and quadratic polynomials to approximate the warpage and determine the best combination of molding process parameters. Zhang et al. [10] and Deng et al. [11] utilized mode-pursuing sampling (MPS) algorithms to minimize warpage. In addition, the single-objective search for Simulated Annealing Optimization (SAO) has been confirmed to be a successful method for finding suitable parameters in the injection molding process [12,13,14,15,16,17]. Researchers such as Dimla et al. [18], Wang et al. [19], Kitayama et al. [20,21,22,23], and Guo et al. [24,25] have empowered related fields by fusing innovative developments to substantially improve the quality and output of goods. The application of such complex computational methods in the context of PIM involves an ensemble of artificial intelligence networks, operations research, and computational physics. Ouyang Yu et al. [26] conducted a five-factor and four-level orthogonal experiment to establish the injection molding process of a funboard. With the maximum warpage deformation, volume shrinkage rate, and shrink mark length as the assessment parameters, the signal-to-noise ratio and normalization were used to process the test results. The multi-objective optimization was converted into a single-objective gray correlation degree (GRA) analysis. Another study (Tan and Alkan [27]) optimized the production efficiency and quality parameters of piezoelectric pumps produced via the microinjection method. An additional investigation was carried out in which the injection molding process parameters were optimized using RSM and GWO methods; it was found that RSM was more efficient, with a 39.4% improvement in the tensile modulus obtained by using 60% fiber reinforcement [28]. Beyond polymer injection molding, the applicability of RSM has also been validated in different material systems. Shetty et al. examined magnesium AZ31B alloy corrosion protection in a hydrochloric acid medium by employing gelatin as an inhibitor and optimizing the process through RSM. Their results demonstrated that RSM effectively captured the interaction between temperature and the inhibitor concentration, achieving a maximum inhibition efficiency of 86% [29]. Building on these optimization-focused studies, another study investigated PP, POM, and PBT polymers for implant manufacturing via injection molding. The simulation results showed that PBT exhibited the lowest deformation (1.09 mm) and shrinkage rate (16.76%), along with a favorable stress response (104.2 MPa), making it the most suitable material for biomedical applications [30]. In addition, Nabudda et al. [31] employed computational fluid dynamics to optimize degradable polylactic acid (PLA)-based coating materials for sustainable wire manufacturing, demonstrating that the injection angle strongly influences flow dynamics, heat transfer, density distribution, and pressure uniformity. Their findings revealed that a 45° injection angle ensured superior uniformity and structural integrity, further underlining the significance of multi-parameter optimization in injection-based manufacturing processes.

This study focused on examining the moldability and thermomechanical properties of the advanced engineering materials PEEK, PEI, and PAI for the MS3102A 16S-1P type multi-pin electrical connector geometry via Moldflow simulation. Experimental design techniques such as Box–Behnken design and Response Surface Methodology (RSM) were used to establish the effects of some important process parameters; namely, the melt temperature, injection time, and mold open time, on critical quality characteristics. In this way, this study takes a very relevant and practical step towards material identification and process optimization for the manufacturing of high-performance electrical components.

## 2. Materials and Methods

### 2.1. Materials

This study tested three types of high-performance engineering plastics—Polyamide-imide (PAI), Polyetherimide (PEI), and Polyether-ether-ketone (PEEK)—which are well known for their thermal resistance and dielectric properties of the highest quality. These materials were picked from the Autodesk Moldflow material database as data suggest that they are the best for manufacturing multi-pin electrical connectors, taking into account their thermal and structural properties. PAI (Torlon^®^ 5030, Drake Plastics, Cypress, TX, USA) is the first choice for areas of application that require the highest accuracy and allow only exposure to heat cycles, because it has a very high glass transition temperature (280 °C), very low water absorption rate (0.4%), and outstanding mechanical strength at high temperatures. It also helps to keep the dimensions stable to the maximum extent by showing the lowest residual stress along the secondary flow direction in the mold. PEI (Ultem™ 1000, SABIC, Riyadh, Saudi Arabia) is an amorphous polymer with a glass transition temperature of about 217 °C and excellent dimensional stability. It is one of the most frequent choices for components of high-voltage technologies because of its reliable electrical insulation efficiency and stability under thermal cycling. The basic material properties are listed in Table 1.

**Figure 1:**
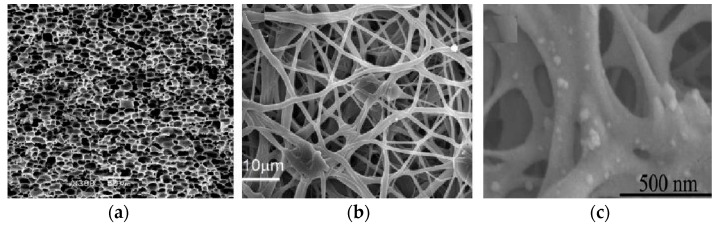
Representative SEM images of (**a**) PAI, (**b**) PEI, and (**c**) PEEK.

PEEK (Victrex^®^ 450G, Victrex, Lancashire, UK) is a typical choice in this context due to its semi-crystalline structure, very high melting temperature of 380 °C, and perfect chemical resistance. At the same time, PEEK retains its mechanical properties at high temperatures in the long-term, but the high crystallinity rate of its structure increases the frequency of molding quality issues, such as volumetric shrinkage and warpage (Figure 1).

### 2.2. Electrical Connector Model

**Figure 2:**
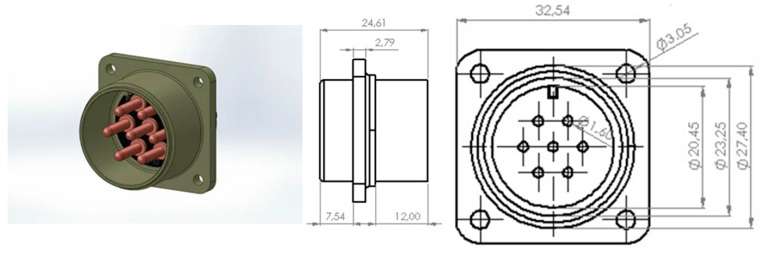
A 3D model and dimensions of the MS3102A 16S-1P circular electrical connector (mm).

This research involved injection molding simulations of the MS3102A 16S-1P model (Mouser Electronics, Mansfield, TX, USA), which is a multi-pin circular electrical connector. Such connectors are the most widely and typically used in the defense and aviation industries for heavy industrial applications. In this context, properties such as high impact resistance, electrical insulation capability, and environmental sealing are the main ones to consider. The model encompasses a total of seven pin cavities, a multi-part cylindrical outer housing, and a volumetric structure that is suitable for centralized injection flow. The part’s geometric properties have a significant impact on the simulation results, especially in the case of temperature homogeneity on the surface and the flow direction inside the cavity. Figure 2 shows a 3D view of the connector model used in the analyses. The part was represented in Autodesk Moldflow Insight 2016 software by using a 3D mesh that was imported in IGS format in order to conduct filling, packing, warpage, and cooling simulations.

### 2.3. Moldflow Simulation Parameters

The simulation studies utilized the Autodesk Moldflow Insight 2016 software. The injection molding process of the electrical connector model was studied, along with a combination of cooling, filling, packing, and warpage simulations. In the simulation model, a 3D tetrahedral (dual-domain) mesh structure was used, having 94,840 surface triangles and 57,380 nodes. A mean aspect ratio of 1.53 allowed for good mesh quality. There were no open edges or intersections found in the geometry. The mesh match percentage was 92.9%, with a reciprocal match of 95.1%. For each material (PAI, PEI, PEEK), independent analysis sets were created, and the results were subjected to multivariate evaluation. In the simulations, the three principal process parameters considered were as follows: melt temperature: 350–390 °C (PAI), 362–402 °C (PEI), or 360–400 °C (PEEK); automatic injection time: 0.095–1 s; and mold open time: 4–6 s. Table 2 shows the general fixed parameters used in the simulations.

### 2.4. Experimental Design and Response Surface Method (RSM)

Through the implementation of a Box–Behnken design (BBD), this investigation aimed to characterize and improve the effects of three principal process parameters—the melt temperature, injection time, and mold open time—on part quality in the injection molding process. BBD is a popular type of RSM that allows the user to generate precise second-order (quadratic) models by performing a small number of experiments. A three-factor, three-level BBD matrix was developed for each material, and Moldflow studies were carried out according to 15 experimental conditions. The design factors and levels are given in Table 3.

In the simulations, six different quality outputs were examined as response variables: volumetric shrinkage (%), sink mark depth (mm), warpage (mm), residual stress (MPa), surface temperature homogeneity (°C), and heat removal efficiency (%). Analysis of variance (ANOVA), contour plot generation, and multi-objective optimization using the Design Expert^®^ v13 software were carried out in this study based on the simulation results. This methodology not only was useful for comparative analyses, allowing both material-based and parameter-based comparisons, but also helped to determine the best processing conditions.

## 3. Results

In the injection molding process, uniform cooling of the polymer inside the mold is essential for dimensional accuracy, residual stress generation, and cycle times. The simulations carried out in this case evaluated the cooling performance, the average surface temperature, and the quantity of heat extracted from the mold for the three polymer materials.

### 3.1. Thermal Performance

When molding high-dielectric-strength polymers, managing the temperature is very important for both structural integrity and dimensional stability. Surface temperature uniformity and heat removal from the cooling phase of the injection molding process are the two main parameters that were studied herein to determine the performance of the part.

#### 3.1.1. Heat Removal Analysis

**Figure 3:**
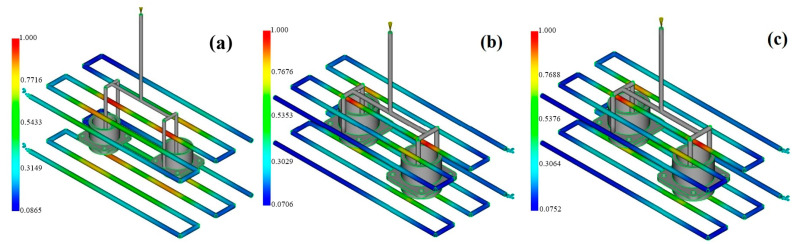
Moldflow heat removal efficiency contours: (**a**) PAI; (**b**) PEI; (**c**) PEEK.

In the injection molding of high-performance engineering polymers for precision components like multi-pin connectors, the thermal stability after molding and geometric accuracy are of tremendous importance. Hence, thermal performance parameters like the total heat transferred from the mold and the uniformity of the surface temperature were studied as key quality indicators (Figure 3).

The simulation results revealed that the PAI material demonstrated the greatest efficiency with respect to the total heat extracted from the mold. In the first part of the study that used PAI, a total heat output of 0.127 kW was realized by two separate cooling circuits. The corresponding values were found to be 0.117 kW for PEEK and 0.181 kW for PEI. Though the total heat removal for PEI was higher, it came from different regions of the mold through separate inlets, which means that the heat was not uniformly transferred to the whole mold. On the other hand, PAI’s high thermal conductivity coefficient (0.29 W/m·K) and high melt temperature (370 °C) made it possible for the heat to be transferred more effectively to the mold walls, thus cooling the sample in a controlled manner. Based on heat conduction according to Fourier’s law, this situation allows PAI to generate a higher heat flux; thus, this material has a competitive edge in thick-walled, high-power-density connector applications.

#### 3.1.2. Surface Temperature Homogeneity

**Figure 4:**
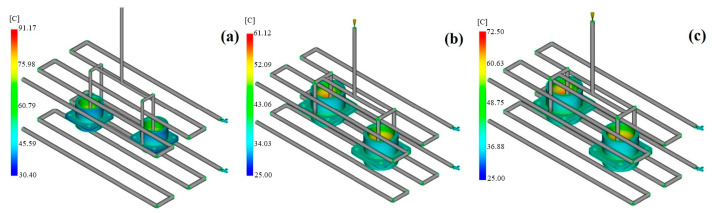
Moldflow surface temperature contours: (**a**) PAI; (**b**) PEI; (**c**) PEEK.

Regarding the homogeneity of the surface temperature, the most balanced thermal distribution was noted for PEI. In cooling studies with PEI, the part’s surface temperature ranged from 31.9 °C to 68.2 °C, with an average of 45.5 °C. The amorphous structure of PEI certainly precludes crystallization-induced orientation effects, thus enabling a more isothermal surface profile. As highlighted in a previous study [32], this feature increases the part’s dimensional stability and reduces the occurrence of shape deformations (Figure 4).

The different materials displayed differences in their cooling behavior. It is very interesting that PAI was processed under the most thermally demanding conditions: a melt temperature of 370 °C. Nevertheless, through its high thermal conductivity, PAI managed a controlled cooling profile, which represents a significant achievement. This shows that PAI is a perfect kind of plastic for industrial electronic components that are used in hot environments. It can also be said that due to the crystalline structure of PEEK, it is necessary to cool it for longer; thus, PEEK is at a disadvantage when a homogeneous temperature distribution is required. Consequently, the simulation data revealed that PAI exhibits the best thermal stability in applications requiring high thermal resistance, while PEI is the best choice for molding operations where there is a need for geometric accuracy and surface temperature constancy.

### 3.2. Structural Stability

During the injection molding process, structural stability plays a very important role for parts with very tight dimensional tolerances—like electrical connectors—not only for the final dimensional conformity, but also for the precision of the electrical contact and the reliability of the mechanical interlock. Particularly in multi-pin connection systems, errors of even a few millimeters directly impact the pins’ alignment accuracy. Here, the warpage and residual stress information obtained from the simulations is discussed from the point of view of the producibility and performance of the part.

#### 3.2.1. Warpage

**Figure 5:**
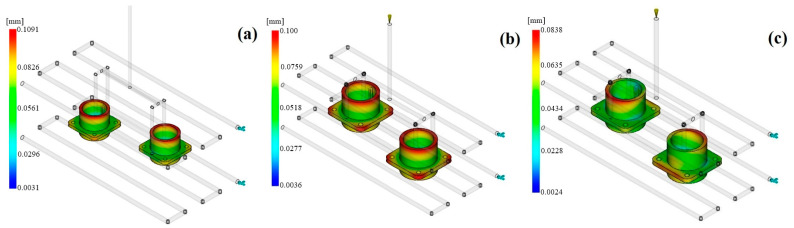
Moldflow warpage contours: (**a**) PAI; (**b**) PEI; (**c**) PEEK.

Warpage describes changes in the size of parts made via the injection molding process and is directly related to the functional reliability of high-precision components such as connectors. When a part has an asymmetric, multi-pin geometry, the stress distributions after molding can be very different; thus, the warpage tendencies can become more visible. To illustrate this, the minimum value of warpage among the three engineering polymers chosen for this research was registered for PAI, at 0.254 mm. Though this figure reflects PAI’s melting temperature, which is very high (370 °C), as well as the temperature of the mold being 160 °C, it still implies that the material can give a more balanced cooling profile all over the mold (Figure 5).

Simulations performed with the PEEK and PEI materials resulted in warpage measurements of 0.279 mm and 0.263 mm, respectively. The semi-crystalline nature of PEEK resulted in a shrinkage difference that was also caused by the orientation of the mold combined with temperature gradients, resulting in increased potential for geometric deformation. Despite the fact that PEI showed more isotropic shrinkage behavior due to its amorphous structure, a warpage effect was still present due to the difference in the surface and core temperatures, caused by the low thermal conductivity of PEI. In this situation, PAI is regarded as the most appropriate option in terms of dimensional stability because of its high modulus and better thermal management ability.

#### 3.2.2. Residual Stress

**Figure 6:**
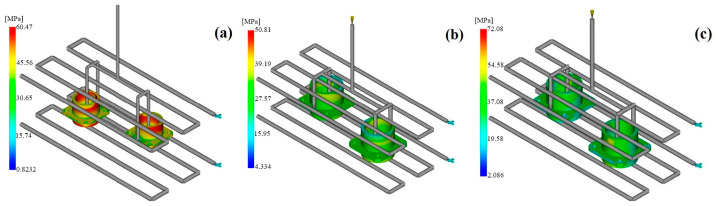
Moldflow residual stress contours: (**a**) PAI; (**b**) PEI; (**c**) PEEK.

Residual stresses that develop within a component after molding are the main factor determining the dimensional stability and mechanical properties over the lifetime of the component. The simulation results showed that PEEK exhibited the lowest peak residual stress (~60 MPa). PAI presented a more uniform residual stress distribution (lower gradient); however, its localized maximum in oriented regions was higher (~81.9 MPa). PEI lay between these two cases (Figure 6).

The high residual stress accumulation in PEEK that was revealed in this research is a result of polymer chains orienting during the crystallization process. Such orientation causes local stress concentrations, especially in narrow cross-sectional areas near the pin entrances of the connector model. Although PEI has no crystallization-induced stress because of its amorphous nature, its low thermal conductivity gives rise to thermal stress differences between the core and surface regions of the part. On the other hand, PAI’s high thermal conductivity and low coefficient of thermal expansion enable the lowest stress gradient to occur during cooling, thus ensuring a more uniform stress distribution. The obtained results demonstrate that PAI ensures not only better dimensional stability but also internal structural homogeneity, so it is most suitable for high-precision connector applications. Hence, the use of PAI in the manufacturing of connectors can be regarded as the most reasonable solution from the points of view of long-term shape stability and assembly reliability. Furthermore, insights from other scales of investigation support the critical role of residual stress in polymer systems. Through molecular dynamics simulations, Rahimian-Koloor and Shokrieh [33] demonstrated that curing-induced residual stresses in carbon nanotube/epoxy nanocomposites reached up to 33% of the applied stress, significantly influencing the overall mechanical response. These findings underline that residual stress is a multi-scale phenomenon, and its impact must be considered not only in injection-molded engineering polymers but also in advanced composite materials.

### 3.3. Mold-Induced Dimensional Deviations

During the injection molding process, deviations caused by the mold have an impact on the geometric accuracy and functional compliance of the final product. Especially in those electrical connectors for which multiple pins and precision tolerance are the main aspects, volumetric shrinkage and sink marks, due to their nature, affect essential functions like pin alignment, locking tolerances, and sealing performance. In this way, the shrinkage phenomenon and surface defects caused by the material’s thermomechanical properties not only affect the visual quality of the component but also change its functional reliability. Moldflow analyses, in this sense, identified the characteristics of dimensional variation of the PAI, PEI, and PEEK polymer materials resulting from different processing parameters. The following subsections present the volumetric shrinkage aspect and sink mark formation for each material in depth.

#### 3.3.1. Volumetric Shrinkage

**Figure 7:**
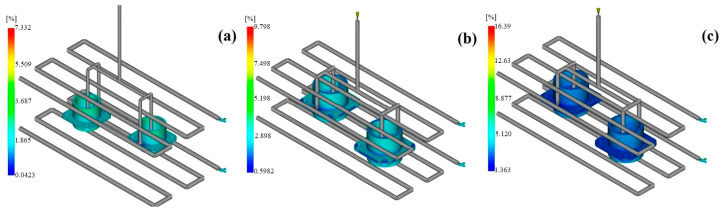
Moldflow volumetric shrinkage contours: (**a**) PAI; (**b**) PEI; (**c**) PEEK.

Volumetric shrinkage is the one of the main factors affecting the dimensional tolerances of a part; it occurs due to cooling of the polymer during the injection molding process and is directly related to the final product’s volume. The examined connector is a model with several pin sockets, thick-walled regions, and different cross-sectional geometries, resulting in different cooling rates in various parts of the mold. Therefore, instead of uniformly contracting as a whole, parts of the component shrink at different rates (Figure 7).

Based on the simulation results, among the three simulated engineering polymers, PEI showed the lowest average volumetric shrinkage rate. In the PEI analyses, the shrinkage rate ranged from 1.03% to 1.41% due to its amorphous structure, which is responsible for isotropic shrinkage behavior. This feature’s main benefit is to support dimensional stability after molding. For PEEK, the volumetric shrinkage ranged from 1.22% to 1.41%. Due to its semi-crystalline nature, PEEK undergoes crystallization-induced shrinkage orientation during the cooling phase, which, in turn, results in dimensional changes, especially in the thick-walled areas. For PAI, the mean shrinkage rate was found to be from 1.09% to 1.29%. The high melting and glass transition temperatures of PAI result in polymer chains that become more compact as they are heated, leading to controlled shrinkage behavior at high temperatures. Even though PAI is not a crystalline phase and has a very dense amorphous structure, it still has to be handled carefully as it has a high processing temperature, similarly to PEEK. Within this framework, PEI can be recognized as the best choice of material for connector applications where dimensional tolerance is essential, due to its low and isotropic shrinkage performance. On the other hand, PEEK and PAI have higher thermomechanical resistance, but they have to be regulated more tightly during processing to shrink as effectively as intended.

#### 3.3.2. Sink Mark Depth

**Figure 8:**
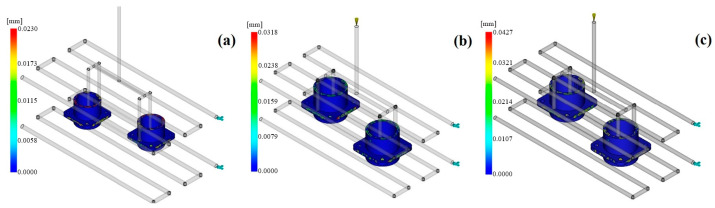
Moldflow sink mark depth contours: (**a**) PAI; (**b**) PEI; (**c**) PEEK.

Sink marks are surface dents caused by material shrinkage during the cooling stage of the injection molding process, usually in thick-walled parts. Besides affecting the surface appearance, these defects can also impair the mechanical integrity and sealing performance of precision components such as electrical connectors. For the connector model examined herein, gatherings of material at pin housings and the places where the local wall thickness changes have been confirmed to be the areas that are most vulnerable to sink mark generation (Figure 8).

According to the Moldflow simulations, PEI demonstrated the smallest sink mark depth. When we compared the models produced with PEI, we found that the sink mark depth was reduced to about 0.018 mm. This result was credited to the low glass transition temperature and slow cooling rate of PEI, which are characteristics consistent with densification of the core regions occurring in a controlled manner. Besides this, elimination of the amorphous structure of PEI caused by the crystallization process induced volumetric orientation, thus reducing the surface deformation. The sink mark depth for the PAI material was in the range of 0.023–0.027 mm. This is because the temperature difference remained intact within the mold for a long time during the process at high temperature and along the material’s solidification curve. PAI’s high thermal expansion coefficient can partly compensate for this due to the principle of energy transfer, but limited energy transfer to the core zones appears in the thick regions after these core zones are filled. The sink mark depth for PEEK was the highest and reached 0.031 mm. PEEK, being a semi-crystalline polymer, exhibits crystallization differences in the core–shell structure. Those differences cause the surface to shrink unevenly, especially in thick-section transitions, such as rib structures and the locking teeth of the connector. In addition, given that its viscosity is high, pressure compensation may not be sufficient in the core zones, which means that the sink marks become even more prominent. It is worth noting here [32] that adjusting the packing pressure, having the same mold temperature, and choosing amorphous materials are some effective methods to reduce sink marks. Thus, PEI is the ideal material, with its smooth surface and integral structure, for connector applications.

### 3.4. Comparative Evaluation

This investigation covered several quality inputs, including heat flux, residual stress, warpage, volumetric shrinkage, sink mark depth, and surface temperature. The outcomes were correspondingly predicted by means of desirability functions. Optimization based on desirability was carried out by applying the Derringer–Suich method, which consists of a step that defines the direction of the target (minimization or maximization) for each response individually to calculate the corresponding desirability value (d_i_). After that, the overall desirability index (D) was obtained for each material.

This technique provides a convenient way of finding the most suitable process parameter intervals that meet the quality requirements for each polymer. The connector’s inner segments were made of PAI, which stands out as a material with high thermal conductivity and good temperature stability. The superior heat flux performance of PAI not only makes it possible to cool the mold efficiently, but also resolves the residual stress problem if the process is controlled properly. Since the trends of shrinkage and deflection stay within acceptable limits, PAI also provides an advantage in terms of dimensional stability. PEI, being an amorphous material as a result of its structure, showed the least amount of warpage and sink marks among the examined materials. Its uniform surface temperature distribution and low residual stress make PEI a perfect choice for outer shell components where it is important to have visual and dimensional quality. At the same time, its process tolerance range remains relatively wide and stable. PEEK may still be a good choice for load-bearing applications that need high mechanical strength, but it is definitely more vulnerable to dimensional defects like shrinkage and sink marks. The fact that PEEK had the lowest residual stress among the three materials means that it would provide long-term structural stability, though the number of process parameters could be narrowed down and optimized.

#### 3.4.1. Desirability Functions and Methodological Validation

In this evaluation, the following targets were defined for each quality output:

The outputs to be minimized: *residual stress, warpage, volumetric shrinkage, sink mark depth.*

The outputs to be maximized: *heat flux.*

The individual desirability function for each output was defined as follows:

$$\begin{array}{c} \;\;\;\{1\;-\;(Y\;-\;L)/(T\;-\;L)\;(Minimum\;target)\\ d_{i}\;=\\ \;\;\;\{(Y\;-\;L)/(T\;-\;L)\;(Maximum\;target) \end{array}$$

The overall desirability score was calculated using the following formula:

D = (d_1_·d_2_·d_3_·d_4_·d_5_)^(1/5)^

Here,

d_1_–d_6_ = desirability functions based on quality responses (values ranging between 0 and 1);

d_7_ = stability-based weighted desirability function;

Y = observed value;

L = acceptable ideal value;

T = worst acceptable value.

This approach is highly consistent with the multi-objective optimization techniques suggested in the literature. In particular, material evaluation research based on RSM by Tan [28] and Shi et al. [8] proved the efficacy of this method for complicated manufacturing operations like injection molding (Table 4 and Table 5).

#### 3.4.2. Response Surface Interactions

The injection molding of high-performance polymers is a complicated and nonlinear process involving several interdependent relationships between process parameters and the outcomes of part quality. To uncover new multivariable interactions, response surface graphs for each material were produced via Box–Behnken design (BBD). These 3D figures visually illustrate the joint influence of the main process parameters—like the melt temperature, injection time, and mold open time—on qualities such as the warpage, residual stress, volumetric shrinkage, and sink mark depth of the part. The developed response surfaces not only open the way for identifying the relationships between parameters and outputs but also confirm the outputs’ sensitivity. Thus, they assist the multi-objective optimization process by facilitating the detection of stable processing windows and possible equilibrium points. The ensuing graphs present these interaction surfaces retrieved for PAI, PEI, and PEEK, enabling engineering-based interpretations of the observed tendencies.

## 4. Conclusions

In this research, the injection molding procedure for MS3102A 16S-1P multi-pin electrical connectors using high-performance engineering polymers—PAI, PEI, and PEEK—was examined in a comprehensive manner through Autodesk Moldflow simulations and the Response Surface Methodology (RSM). The number of simulations was 45, wherein 15 different process combinations were tested for each material set according to the Box–Behnken experimental design. The main quality outputs observed were the heat flux removed from the mold, residual stress, warpage, volumetric shrinkage, and sink mark depth. The following results were obtained from the multi-objective optimization analysis based on the Derringer–Suich desirability function:

- The PEI material showed great potential with respect to surface quality and dimensional accuracy, with a sink mark depth of 0.018 mm, volumetric shrinkage of 1.03–1.41%, and deflection of 0.263 mm. Although its thermal conductivity is lower than that of PAI, the amorphous structure of PEI promotes isotropic shrinkage and suppresses crystallization-induced orientation, leading to more uniform volumetric contraction. This characteristic explains why PEI achieves superior dimensional stability despite its lower heat transfer capability. Due to the fact that it can create an isothermal profile in the surface temperature distribution, it is the best option for outer shell parts.
- The PAI material demonstrated better thermal management performance than other materials, giving the best heat flux value of 0.127 kW. Although PEI exhibited a higher total heat removal value (0.181 kW), the distribution was less homogeneous, resulting in localized hot spots. PAI, despite its lower total heat removal (0.127 kW), provided a more uniform heat flux across the part, which is more favorable for dimensional stability and residual stress reduction. Its deflection (0.254 mm) and shrinkage (1.09–1.29%) were still within acceptable limits, but the residual stress value (81.9 MPa) is problematic, as acceptable limits for connector applications are typically in the range of ~50–80 MPa (≈25–35% of tensile strength). This material is definitely the best option for internal conductive parts operating at high temperatures.
- While PEEK exhibited the lowest peak residual stress (~60 MPa), it showed the weakest performance in terms of dimensional stability (0.279 mm) and surface quality, having a shrinkage rate of 1.22–1.41% and a sink mark depth of 0.031 mm. By contrast, PAI showed a more uniform distribution but a higher localized maximum (~81.9 MPa), with PEI presenting intermediate behavior. However, PEEK can be regarded as a good candidate for structural support applications (e.g., anchoring regions) due to its high mechanical load-bearing capacity and low deflection tendency. In addition to these thermal and structural evaluations, the findings are also relevant to dielectric performance. Although dielectric strength was not directly simulated, the results indicate that uniform cooling and lower residual stress contribute to reducing microvoids and localized defects, which are critical factors for dielectric breakdown. Consistent with the material data in Table 1, PAI, with the highest dielectric strength (25 kV/mm), exhibited superior thermal management, while PEI (20 kV/mm) provided the most homogeneous surface temperature distribution. These correlations strengthen the relevance of this study to dielectric reliability in high-performance connector applications.

Taking these results into account, the suggested best process ranges and use cases for each material are presented below.

Although the recommended melt temperature ranges in Table 6 partly overlap, the simulations revealed that sensitivity to deviations outside these ranges differed significantly among the polymers. PEEK was the most sensitive: below the lower bound, its viscosity increased and short-shot risk was observed, while above the upper bound, crystallization-driven shrinkage gradients caused higher warpage and sink marks. PAI showed moderate sensitivity, with lower temperatures increasing its sink-mark tendency and higher temperatures leading to elevated peak residual stress. PEI exhibited the lowest sensitivity, where small deviations produced only minor effects on pressure and warpage, though higher melt temperatures prolonged cooling without major quality benefits. The response surface functions, with model accuracy coefficients at the R^2^ > 0.95 level, have sufficient statistical power in explaining the interactions between process parameters and quality outputs. This study shows that injection molding is not only a matter of choosing appropriate materials, but also a matter of detailed process parameter optimization that determines the product quality. The Moldflow-based RSM approach serves as a decision support tool for the high-quality and reliable production of functionally complex and engineering-driven electrical connectors. To enhance the reliability of the simulation results, experimental validation under real service conditions would be highly appropriate. For multi-pin connectors in particular, ensuring long-term performance and reliability requires careful implementation of thermal cycling, dielectric breakdown, moisture resistance, and mechanical loading tests. Such experimental validation would not only confirm the accuracy of the simulation predictions but also provide deeper insights into the interactions between processing parameters and material morphology, thereby contributing to more robust engineering approaches in connector design. Future research could determine the influence of cooling channel configurations on the molding process, comparatively analyze fiber-reinforced variants, and carry out model validation by means of post-molding dimensional measurements of physical prototypes. This would cover some significant gaps in the present academic literature.

## Figures and Tables

**Table 1:** Basic physical and thermal properties of polymer materials.

Properties	Unit	PAI	PEI	PEEK
Melt Temperature	°C	370	382	380
Glass Transition Temperature	°C	280	217	243
Water Absorption	24 h, %	0.4	0.5	0.1
Solid Density	g/cm^3^	1.41	1.27	1.3
Thermal Conductivity	W/m·K	0.29	0.22	0.25
Dielectric Strength	kV/mm	25	20	20

**Table 2:** Fixed parameters in Moldflow simulations.

Parameter	Unit	PAI	PEI	PEEK
Mold Temperature	°C	160	160	160
Ambient Temperature	°C	25	25	25
Injection Pressure Limit	MPa	180	180	180
Mesh Type	-	3D	3D	3D
Average Element Count	-	95,000	95,000	95,000
Cooling Analysis Type	-	Automatic	Automatic	Automatic

**Table 3:** Process parameters and level ranges used in the experimental design (PAI).

Input Parameter	Unit	Symbol	Low	Medium	High
Melt Temperature	°C	A	350	370	390
Injection Time	s	B	0.095	0.0975	1
Mold Open Time	s	C	4	5	6

**Table 4:** Desirability functions by output.

Output	Target	Desirability Function (dᵢ)
Volumetric Shrinkage	Minimum	d_1_ = 1 − (Y − L)/(T − L)
Residual	Minimum	d_2_ = 1 − (Y − L)/(T − L)
Warpage	Minimum	d_3_ = 1 − (Y − L)/(T − L)
Heat Flux	Maximum	d_5_ = (Y − L)/(T − L)
Sink Mark Depth	Minimum	d_6_ = 1 − (Y − L)/(T − L)

**Table 5:** Desirability comparison of polymer materials.

Quality Output	Target	PAI	PEI	PEEK
Heat Flux	Maximum	**🟢** High	**🔴** Low	**🟡** Medium
Residual Stress	Minimum	**🔴** High	**🟡** Medium	**🟢** Low
Warpage	Minimum	**🟡** Medium	**🟢** Low	**🟡** Medium
Volumetric Shrinkage	Minimum	**🟡** Medium	**🟢** Low	**🔴** High
Sink Mark Depth	Minimum	**🟡** Medium	**🟢** Low	**🔴** High

**🔴**: Poor Desirability; **🟡**: Acceptable; **🟢**: Desirable.

**Table 6:** Processing parameters and application types.

Material	Optimum Melt Temp (°C)	Mold Open Time (s)	Application Type
PAI	370–380	6 (max)	Inner conductive regions operating at high temperatures
PEI	370–380	5–6	Dimensionally precise outer casings with visible surfaces
PEEK	360–370	6 (max)	Mechanically reinforced anchoring zones

## Data Availability

The raw data supporting the conclusions of this article will be made available by the author on request.

## Abbreviations

The following abbreviations are used in this manuscript:

RSM	Response Surface Methodology
BBD	Box–Behnken design
PAI	Polyamide-imide
PEI	Polyetherimide
PEEK	Polyether-ether-ketone
PIM	Plastic injection molding
